# Interaction of ncRNAs and the PI3K/AKT/mTOR pathway: Implications for osteosarcoma

**DOI:** 10.1515/biol-2022-0936

**Published:** 2024-08-06

**Authors:** Weilin Shao, Yan Feng, Jin Huang, Tingyu Li, Shengguai Gao, Yihao Yang, Dongqi Li, Zuozhang Yang, Zhihong Yao

**Affiliations:** Bone and Soft Tissue Tumours Research Centre of Yunnan Province, Department of Orthopaedics, The Third Affiliated Hospital of Kunming Medical University (Yunnan Cancer Hospital, Yunnan Cancer Center), Kunming, Yunnan, 650118, China; Clinical Oncology Institute, Kunming Medical University, Kunming, Yunnan, 650500, China; Bone and Soft Tissue Tumours Research Centre of Yunnan Province, Department of Orthopaedics, The Third Affiliated Hospital of Kunming Medical University (Yunnan Cancer Hospital, Yunnan Cancer Center), No. 519 Kunzhou Road, Xishan District, Kunming, Yunnan, 650118, China; Department of Cancer Research Institute, The Third Affiliated Hospital of Kunming Medical University (Yunnan Cancer Hospital, Yunnan Cancer Center), No. 519 Kunzhou Road, Xishan District, Kunming, Yunnan, 650118, China

**Keywords:** osteosarcoma, PI3K/AKT/mTOR, ncRNAs, biomarkers, therapeutic targets

## Abstract

Osteosarcoma (OS) is the most common primary malignant bone tumor in children and adolescents, and is characterized by high heterogeneity, high malignancy, easy metastasis, and poor prognosis. Recurrence, metastasis, and multidrug resistance are the main problems that limit the therapeutic effect and prognosis of OS. PI3K/AKT/mTOR signaling pathway is often abnormally activated in OS tissues and cells, which promotes the rapid development, metastasis, and drug sensitivity of OS. Emerging evidence has revealed new insights into tumorigenesis through the interaction between the PI3K/AKT/mTOR pathway and non-coding RNAs (ncRNAs). Therefore, we reviewed the interactions between the PI3K/AKT/mTOR pathway and ncRNAs and their implication in OS. These interactions have the potential to serve as cancer biomarkers and therapeutic targets in clinical applications.

## 1 Introduction

In pediatric and adolescent populations, osteosarcoma (OS) is a primary malignant bone tumor that often arises in the metaphysis of the long bone [1]. The incidence of OS varies according to sex, age, and race. The annual incidence of OS is approximately three per million [2]. Clinically, adolescents aged 10–20 years are at high risk of OS. Studies have shown that people above the age of 50 years have the second highest OS-associated morbidity [3]. Approximately 15–20% of patients with OS are diagnosed with clinically detectable distant metastases, with over 85% of these metastases occurring in the lungs [4]. With the clinical application of neoadjuvant chemotherapy, the 5-year survival rate of patients with OS has increased to 70% [5]. However, the 5-year survival rate of OS patients with metastasis remains below 20% [6]. Currently, standard treatments for patients with OS are preoperative neoadjuvant chemotherapy, surgery, and postoperative adjuvant therapy [7]. However, the lack of targeted drugs is the clinical bottleneck of the treatment for the patients with OS [8]. The unknown etiology, recurrence, metastasis, multidrug resistance, and extensive histological specificity of OS obstructs the treatment procedure of patients with OS. Thus, it is urgent to study the pathogenesis and metastasis of OS.

Substantial evidence suggests that the imbalance of multiple signaling pathways is closely related to the progression of OS; while, PI3K/AKT/mTOR pathway plays a unique role in the progression and clinical treatment of OS [9]. The PI3K/AKT/mTOR signaling pathway plays a crucial role in promoting tumor cell proliferation, migration, epithelial–mesenchymal transition (EMT), inhibiting cell apoptosis, and increasing sensitivity to chemotherapy drugs [10]. Interestingly, the PI3K/AKT/mTOR signaling pathway is widely activated in the tissues and cells of OS. Overactivation of this pathway has oncogenic effects. Thus, PI3K/AKT/mTOR signaling inhibitors to block the activation of effector molecules and promote apoptosis have become a new idea for the clinical treatment of OS [11]. However, the biological functions and specific regulatory mechanisms of the PI3K/AKT/mTOR signaling pathway are poorly understood, and many problems still need to be further studied.

Some non-coding RNAs (ncRNAs) can act on certain steps of this pathway, regulating its activity and thereby affecting the progression of tumors [12,13]. These ncRNAs can generally be divided into two categories based on their effects on tumor cells. Those that are overexpressed in OS tissues, enhance tumor progression or drug resistance by promoting PI3K/Akt/mTOR pathway signaling; while, those that are downregulated in tumor tissues, inhibit tumor progression by inhibiting the PI3K/Akt/mTOR pathway. By studying the molecular mechanisms of various ncRNAs, we can explore the mechanisms of OS development, metastasis, or drug sensitivity. Based on this theoretical foundation, we reviewed the interactions between the PI3K/AKT/mTOR pathway and ncRNAs and their implication in OS. We hope these potential targets can be applied to clinical practice in the future.

## 2 PI3K/Akt/mTOR signaling pathway in oncology

### 2.1 Phosphatidylinositol 3 kinase (PI3K)

PI3K is categorized as a group of lipid kinases responsible for phosphorylating the 3ʹ-hydroxyl groups of phosphatidylinositol and phosphoinositide [14]. The primary product of this reaction is phosphatidylinositol-3,4,5-triphosphate (PIP3), which is a crucial secondary messenger. PIP3 helps Akt initiate signaling pathways associated with growth, proliferation, and cell survival [15]. According to its different structural features and substrate preference, PI3K is classified into three categories (Ⅰ–Ⅲ) [14]. Different types of PI3K serve various purposes in cell signal transduction, and their subtypes perform different tasks in cell signal transduction. Among all PI3Ks, class I PI3K molecules have been the most frequently studied because they are most closely related to cancer. Class I PI3K consists of a catalytic subunit (p110) weighing 110 kDa and a regulatory subunit (p85) that forms heterodimers [16]. Mammals produce four variants of the p110 isoform (the α, β, γ, and δ isoform) through different genes. Class I PI3K molecules can be found in all types of cells, with high concentrations of p110δ and p110γ in leukocytes [17]. Under basal conditions, the regulatory subunits interact with the p110 catalytic subunits, thereby securing PI3K protein heterodimers. This interaction not only inhibits kinase activity but also guides PI3K toward upstream regulators for activation [18]. Under physiological conditions, extracellular signals typically activate PI3K. Various stimuli can trigger PI3K activation. For example, growth factors such as epidermal growth factor (EGF), platelet-derived growth factor, and insulin-like growth factor (IGF-1) [19] adhere to the N-terminal extracellular region of their specific transmembrane receptor tyrosine kinases (RTKs). This binding leads to the autophosphorylation of tyrosine residues within the cytoplasmic regions of RTKs and associated linker molecules. Subsequently, PI3K is recruited to RTKs via the interface of p85 SH2 territories with phosphorylated tyrosine residues present in the components of the RTK complex. This interaction triggers the allosteric activation of PI3K [20]. In addition to RTKs, G protein-coupled receptors constitute another important class of traditional upstream regulators of PI3K activation. Additionally, small GTPases such as Ras and RAB5 can stimulate PI3K activation both directly and indirectly [19].

### 2.2 Protein kinase B (AKT)

Serine/threonine kinase AKT encompasses a family that includes AKT1, AKT2, and AKT3 in mammals and serves as a crucial mediator of PI3K signaling. To activate PI3K/AKT pathway, AKT and its upstream kinase, 3-phosphoinositide-dependent protein kinase-1 (PDK1) is required for inner cell membrane. This recruitment facilitates the phosphorylation of AKT at Thr308 in the provocation T-loop by PDK1 [21]. The regulatory hydrophobic domain of AKT contains a Ser473 site that can be phosphorylated by mTOR complex 2 (mTORC2) to achieve optimal activation [22]. Activated phosphorylated AKT subsequently moves out from the cellular membrane and phosphorylates numerous downstream substrates, thereby executing AKT functions [23].

### 2.3 Mammalian target of rapamycin (mTOR)

mTOR is a paramount protein found in various organisms, occupying a key position in the growth and metabolism of cells [24]. mTOR is a protein kinase belonging to the PI3K-related kinase (PIKK) family and is primarily associated with regulatory processes such as cell growth, division, metabolism, and survival [25]. mTOR functions are primarily mediated by mTORC1 and mTORC2, each of which exert different effects on cell physiology and metabolism [26]. mTORC1 predominantly regulates cell growth and metabolism by sensing nutritional and energy states and controlling protein synthesis, cell growth, autophagy, and lipid metabolism [27]. mTORC1 is also involved in responses to changes in oxygen and energy levels [28]. In contrast, mTORC2 is associated with cell survival and the organization of cellular cytoskeletal structures. mTORC2 is vital in insulin signal transduction, influencing lipid metabolism [29]. Disordered activation of the mTOR pathway has been implicated in various diseases, particularly cancers, metabolic disorders, and neurodegenerative diseases [30–32]. Consequently, inhibitors targeting the mTOR pathway, such as rapamycin and its derivatives, are used clinically as anticancer agents and immunomodulators [33]. In summary, mTOR is a fundamental element of cellular physiology and diseases that safeguards human health. Owing to its diverse roles in various diseases, the mTOR pathway has become a hotspot in drug development.

With the deepening of scientific research, the mechanisms of the PI3K/Akt/mTOR signaling pathway are gradually being understood. In human cells, activation of the PI3K pathway starts with RTKs, which are activated upon ligand binding and subsequently activate PI3K, initiating its activation. Activated-PI3K promotes the phosphorylation of phosphatidylinositol-4,5-bisphosphate (PIP2) to generate phosphatidylinositol-3,4,5-trisphosphate (PIP3). PIP3 attracts proteins with PH domains, including Akt. Upon recruitment to the cell membrane, Akt undergoes phosphorylation by PDK1 and mTORC1, becoming p-Akt. p-Akt can inhibit tuberous sclerosis complex 2 (TSC2), which in turn hydrolyzes Rheb-GTP to Rheb-GDP. Rheb-GTP, a GTPase, directly activates mTORC1. Through a series of actions, p-Akt promotes the activation of mTORC1, which then influences cellular activities directly through its own actions [23]. In patients with tumor, the PI3K/Akt/mTOR signaling pathway often exhibits overactivation [34], primarily due to dysregulation in the expression of PIK3CA and phosphatase and tensin homolog (PTEN). PIK3CA encodes p110α, and its increased expression can lead to overactivation of the PI3K pathway [35]. PTEN is a tumor suppressor gene that encodes a phosphatase capable of dephosphorylating PIP3 to PIP2, thereby inhibiting the PI3K pathway. In patients with OS, loss of the PTEN gene is common, which is an important mechanism leading to overactivation of the PI3K pathway [36].

## 3 Interaction of ncRNAs and the PI3K/AKT/mTOR pathway in oncology

ncRNAs do not possess the ability to directly translate into proteins. However, they serve as essential transcripts for regulating the expression of functional genes [37,38]. Based on their nucleotide length, ncRNAs are categorized into various species, mainly including miRNAs, lncRNAs, circRNAs, snRNAs, rRNAs, and tRNAs [39]. With the advancing research in epigenetics, the importance of ncRNAs has gradually been recognized. Experiments by Yu et al. demonstrated that miR-4524b-5p attenuates the progression of glioblastoma (GBM) by targeting aldehyde dehydrogenase ALDH1A3, thereby inhibiting proliferation and radio-resistance through the PI3K/AKT/mTOR signaling pathway [40]. MiR-4524b-5p acts as a targeted inhibitor of ALDH1A3, reducing tumor cell glycolysis and lowering the activation of the PI3K pathway, thus inhibiting GBM progression. Research by Gao et al. indicated that circ-NIRP1, overexpressed in biliary tract cancer (BTC), promotes carcinogenesis by competitively inhibiting miR-515-5p. This inhibition indirectly upregulates Akt2 and activates the PI3K/Akt/mTOR pathway, promoting BTC proliferation, EMT, and stemness [41]. Overall, the interactions between ncRNAs and signaling pathways are increasingly recognized. However, due to their diversity and complex connections, deciphering their associations presents challenges. Consequently, we summarized some common interactions between ncRNAs and the PI3K/AKT/mTOR pathway in OS. Multiple critical regulators originating from the PI3K/AKT/mTOR pathway inter-regulate and activate with ncRNA, resulting in their involvement in cancer progression. Alternatively, ncRNAs can mediate downstream targets to reciprocally affect the expression of key proteins through the PI3K/AKT/mTOR pathway. Therefore, additional discussions regarding the interaction between PI3K/AKT/mTOR and ncRNAs offer insights into the mechanism of malignant behavior in OS.

### 3.1 Long non-coding RNAs (lncRNAs) in oncology

The length of lncRNAs exceeds 200 base pairs, and studies have shown that they participate in the regulation of tumor growth and metastasis [42]. The expression of lncRNAs in the human body plays a crucial role in regulating cell replication cycles, apoptosis, and impacting cell proliferation and migration [37,43,44]. Aberrant expression of lncRNAs can disrupt these cellular processes, contributing to uncontrolled cell proliferation and migration, which are foundational to cancer development. Furthermore, lncRNAs are implicated in chemotherapy resistance; dysregulated expression is linked to reduced efficacy of chemotherapeutic drugs such as doxorubicin [45], and in another context, lncRNAs are associated with resistance in triple-negative breast cancer [46]. As research on lncRNAs advances, they are widely recognized as functional competitors of miRNAs, influencing cellular processes [47]. Researchers believe that developing clinical applications targeting lncRNA mechanisms is feasible. LncRNAs are considered potential targets for prostate cancer therapy [48], and similar studies are also emerging in OS research. LncRNA H19 is overexpressed in OS cells and shows substantial differences compared to its expression in normal cells. Knockdown experiments established that the expression grade of lncRNA H19 is positively correlated with distant metastasis in OS. LncRNA H19 further worsens the condition in patients with OS. LncRNA H19 promotes the phosphorylation of PI3K and Akt and affects the escalation of OS by activating the NF-κB pathway [49]. LncRNA00968 levels are higher in OS cells than in normal and can promote tumor cell survival and colony formation. Knockdown experiments have shown that lncRNA00968 can target and promote the PI3K/AKT/mTOR pathway by acting as an oncogene [50]. The level of lncRNA RUSC1-AS1 in OS cells was higher than that in human osteoblast cell lines. Gene knockout experiments confirmed that lncRNA RUSC1-AS1 enhanced the amplification and extension of OS cells and stimulated EMT. This effect may be achieved through direct targeting of miR-340-5p and indirect activation of the PI3K/Akt pathway [51]. In OS cells, lncRNA-p21 levels are lower. According to the cell growth curve and colony formation experiments, lncRNA-p21 had an inhibitory effect on tumor cells. Overexpression experiments demonstrated that lncRNA-p21 upregulated PTEN by targeting the oncogene miR-130b. Upregulation of PTEN inhibits Akt phosphorylation and suppresses Akt pathway signaling [52]. However, some lncRNAs have the opposite mechanism. In OS cells, LncRNA UCA1 shows upregulation. Luciferase and chromatin immunoprecipitation have shown that the upregulation of UCA1 may be related to hypoxia inducible factor-1 (HIF-1α). HIF-1α interlocks with the responded elements in the UCA1 promoter region, inducing UCA1 expression and downregulating PTEN, activating the Akt pathway, and inducing tumor cell growth [53]. Furthermore, there may be a carcinogenic pathway for UCA1. Through CREB1-mediated PI3K/Akt/mTOR signaling, UCA1 upregulates the expression of CREB1, competitively binds miR-582, and accelerates EMT pathway, thereby boosting tumor metastasis [54]. A positive feedback loop can be formed in OS cells when lncRNA TUG1 is elevated by FOXM1, which competitively absorbs miR-219a-5p. This triggers the Akt pathway, which in turn upregulate the expression of FOXM1, hence boosting the expression of TUG1 [55]. The lncRNA FER1L4 is downregulated in OS and constrains tumor occurrence. In FER1L4 overexpression experiments, a decrease in the expression of the amino acids Ser 470 and Thr 308 constituting p-Akt was detected, indicating that FER1L4 inhibits the phosphorylation of Akt. MiRNA-18a-5p is thought to be the target of lncRNA FER1L4, which suppresses miRNA-18a-5p to prevent the activation of the PI3K/AKT signaling pathway [56]. According to the research of Li et al., the lncRNA NNT-AS 1 is upregulated in OS tissues. Overexpression experiments have demonstrated that the lncRNA NNT-AS 1 can improve the capacity for invasion, proliferation, and lifespan of U2 OS cells, and the expression level of NNT-AS 1 is negatively correlated with miR-320a, suggesting that NNT-AS 1 may target miR-320a and downregulate its expression, thereby activating Akt and promoting progression of OS [57]. LncRNAs can also affect the prognosis of OS, and certain lncRNAs can be utilized as tumor markers in clinical applications. Compared with normal cell types, OS cell lines exhibit much greater expression of the recently identified oncogenic RNA lncRNA LOXL1-AS1. Functional studies have shown that LOXL1-AS1 knockout inhibits the PI3K/AKT pathway, which in turn prevents OS proliferation and invasion. Therefore, in the tissues of patients with OS, the high expression of LOXL1-AS1 plays the role of an oncogene and indicates a poor prognosis [58]. Jiang et al. [59] revealed a strong correlation between tumor size and metastasis in patients with OS and overexpression of lncRNA DANCR. The reason for this correlation is that DANCR binds to miR-33a-5p in a competitive manner, thereby increasing the expression of RTK AXL. This interaction influences the expression of downstream proteins in the PI3K/Akt pathway and affects various aspects of tumor cells, such as the self-renewal of CSC and EMT. Consequently, DANCR was deemed a self-sufficient prognostic element, indicating an unfavorable prognosis for patients with OS.

Similarly, the expression of the lncRNA ANRIL performs an essential function in the prognostic prediction of OS sufferers [60]. Experimental evidence has demonstrated that ANRIL expression in OS tissues is notably higher than that in adjoining non-cancerous tissues. ANRIL enhances the proliferation and invasion of OS cells, and its knockdown appreciably induces mobile apoptosis, confirming its association with a negative prognosis for OS. Specifically, this could also be due to a decrease in the phosphorylation tiers of PI3K and Akt after ANRIL knockdown, leading to subsequent signaling cascade reactions.

In addition to influencing the occurrence and prognosis of OS, lncRNAs affect the resistance of OS to chemotherapeutic drugs. The lncRNA OPI5-AS1 is upregulated in OS MG-63 and Sao-S 2 cells and regulates tumor cellphone resistance to cisplatin. Dual-luciferase reporter gene assays have located that lncRNA OPI5-AS1 can absorb miR-340-5p and regulate the goal lysophosphatidic acid acyltransferase β (LPAATβ) of miR-340-5p. LPAATβ can deactivate the PI3K/AKT pathway. In summary, lncRNA OPI5-AS1 may activate the PI3K/AKT pathway via focused on miR-340-5p to upregulate LPAATβ, improving tumor resistance to cisplatin [61].

In addition to cisplatin resistance, OS’s resistance in OS remains a serious issue. These results suggest that lncRNA PVT1 contributes to chemotherapy resistance in OS cells [62]. Comparative studies of cells overexpressing lncRNA PVT1 after gemcitabine treatment and lncRNA PVT1-depleted cells revealed that lncRNA PVT1 promoted proliferation and anti-apoptotic capabilities, whereas lncRNA PVT1 depletion intensified apoptotic tendencies. Subsequent experiments indicated that lncRNA PVT1 downregulates the levels of miR-152. Notably, lncRNA PVT1 induces the activation of the PI3K/Akt, an effect that can be counteracted by miR-152 and its mimic. This series of experimental results demonstrated that lncRNA PVT1 can activate the PI3K/Akt pathway by downregulating miR-152, thereby increasing OS resistance to gemcitabine.

In summary, the roles of lncRNAs in OS vary depending on their different mechanisms.

### 3.2 MicroRNAs (miRNAs) in oncology

miRNAs are a type of ncRNAs that are approximately 22 nucleotides long and are transcribed from endogenous genes. Their structure is single-stranded, and their primary role in organisms is to engage in post-transcriptional management of gene expression [63]. In recent years, as science and technology have progressed, there has been a growing recognition that the expression of miRNAs is associated with the occurrence of many cancers. miRNAs have the potential to feature either as tumor suppressors or promoters through the inhibition of oncogenes’ or tumor suppressor genes’ mRNA [64], respectively, and are also related to tumor metastasis and drug resistance [65]. Many studies have shown that miRNAs contribute to the pathogenesis of breast cancer [66], renal cancer [67], gastric cancer [68], thus attracting attention and OS. Qi et al. [69] employed a co-culture system comprising human bone marrow stem cells and OS cells to examine the effect of mesenchymal stem cells (MSC)-derived exosomes on OS cells. They found that these exosomes considerably promoted OS proliferation and invasion. Subsequent investigations unveiled an overexpression of miR-21-5p in MSC, targeting PIK3R1, which encodes the p85α subunit that regulates PI3K by binding to its p110 subunit. miR-21-5p initiates PI3K/Akt/mTOR signaling by inhibiting PIK3R1. Wang et al. [70] discovered that miR-384 was reduced in OS cell lines and functioned as a tumor suppressor gene. Overexpression of miR-384 significantly reduced the phosphorylation of PI3K and Akt, inhibited proliferation and invasion of MG63 cells, and accelerated apoptosis. Stem-loop binding protein (SLBP) is a target of miR-384, which regulates its expression. SLBP knockout undermined the tumor-promoting effect of miR-384 silencing in OS cell lines, indicating that downregulation of miR-384 promotes tumor growth by upregulating SLBP and activating the PI3K/Akt pathway. Qi et al. [71] found that miR-29a-3p was downregulated in OS cell lines. Overexpression of miR-29a-3p hinders the proliferation and invasion of OS cells and supports apoptosis and autophagy. Thus, it is considered a tumor suppressor. IGF1 activates the IGF1R/PI3K/Akt pathway to promote the occurrence and progression of OS. miR-29a-3p inhibits this phenomenon, thus exerting a tumor-suppressive effect. Liu et al. [72] discovered that overexpression of miR-342-5p substantially inhibited the proliferation and invasion of OS cells, enhanced apoptosis, and increased sensitivity to doxorubicin. Wnt7b is upregulated in OS cell lines and assists in tumor cell proliferation, possibly by activating mTOR C1 via PI3K/Akt. MiR-342-5p targets Wnt7b and inhibits its expression, thus serving as a tumor suppressor. Wang et al. [73] discovered that miR-485-3p was downregulated in OS cell lines. Dual-luciferase assays verified that miR-485-3p may want to directly bind to Akt3 mRNA, inhibiting the Akt/mTOR pathway in tumor cells, thus inhibiting cellular glycolysis, proliferation, and invasion, and acting as a tumor suppressor. Jin et al. [74] found that miR-1224-5p is downregulated in OS tissues. Overexpression of miR-1224-5p targets PLK1, reduces the phosphorylation of PI3K, Akt, and mTOR, and negatively regulates the PI3K/Akt/mTOR pathway, and inhibits tumor growth and EMT. Ru et al. [75] demonstrated that miR-564 was downregulated in patients with OS. Overexpression of miR-564 directly targets Akt, inhibits its transcription and translation, and suppresses tumor cell glycolysis, thereby inhibiting cell proliferation by reducing cellular activity. Xu et al. [76] demonstrated that the expression level of miR-149-5p was remarkably downregulated in OS. TNFRSF12A, also known as FN14, is a member of the tumor necrosis factor (TNF) receptor superfamily and a direct target of miR-149-5p. MiR-149-5p inhibited TNFRSF12A. TNFRSF12A, when bound to its ligand TNF-like weak inducer of apoptosis (TWEAK), can activate multiple signaling pathways, thereby affecting the tumor microenvironment and promoting tumor cell survival and migration. MiR-149-5p inhibits the PI3K/Akt pathway by suppressing TNFRSF12A. Liu et al. [77] showed that heat shock protein 90 (HSP90) can stabilize the phosphorylation state of Akt, and miR-485-5p can target both HSP90 and Akt, inhibiting Akt’s translation and phosphorylation, thereby inhibiting the pathway and OS progression.

Some miRNAs also possess a function similar to that of lncRNAs, affecting chemotherapy resistance in OS. Meng et al. [78] indicate the engagement of miRNA-22 in the evolution of cisplatin resistance. miR-22 was shown to inhibit the proliferation of MG63/CDDP cells both *in vitro* and *in vivo*, thereby enhancing their resistance to cisplatin-induced proliferation. miR-22 can reduce the translation and phosphorylation of PI3K, Akt, and mTOR, thereby decreasing cisplatin-induced autophagy. However, miR-22 also promotes apoptosis in tumor cells and enhances their sensitivity to cisplatin.

In summary, current research on the relationship between miRNAs and tumors continues to deepen.

### 3.3 Circular RNAs (circRNAs) in oncology

circRNAs were discovered in 1976, formed by precursor mRNA by means of back-splicing, connecting the 3ʹ splice web page to the 5ʹ splice site [79]. With recent advances in research, circRNAs have been recognized to possess unique effects on malignant tumors. circRNAs exhibit varied expression patterns across diseases and contribute to the pathogenesis of these conditions [80]. Li et al. [81] indicated that hsa-circ-0006101 (commonly recognized as circ-ORC2) is upregulated in the cytoplasm of OS cells and orchestrates miR-19a expression through direct binding. miR-19a negatively regulates its downstream target PTEN. PTEN downregulation weakens the inhibition of Akt phosphorylation, activates PI3K/Akt, and promotes the proliferation and invasion of OS. Similarly, Liu et al. [82] illustrated that circROCK1 expression is lower in OS and sponges miR-532-5P, causing its expression to decrease. Conversely, downregulation of miR-532-5P promotes PTEN expression, playing a cancer-suppressive role through a similar mechanism. Li et al. [83] demonstrated that silencing circ-0001785 leads to the downregulation of the phosphorylated forms of the three proteins PI3K, Akt, and mTOR, with little change in their total expression, suggesting that circ-0001785 can directly inhibit the phosphorylation of PI3K, Akt, and mTOR, thus suppressing the activity of the PI3K/Akt/mTOR pathway and affecting apoptosis. In accordance with a study by Yang et al. [84], circ-001422 was found to be increased in OS cells and directly sponge miR-195-5p, leading to the upregulation of its downstream target fibroblast growth factor 2 (FGF2), activation of the PI3K/Akt pathway, and promotion of cancer progression while inhibiting apoptosis. Zhang et al. [85] showed that silencing circRNA-CIRH1A significantly increased the level of miR-1276, damaging the activation of the PI3K/Akt pathway and limiting the proliferation of U20 and MG63 cells. Liu et al. indicated that circRNA-103801 is increased in OS cell lines and is involved in the PI3K/Akt pathway, thereby affecting the growth abilities of OS [86]. Li and Li showed that hsa-circ-0007534 in OS can enhance the phosphorylation of Akt and the subsequent activation of cell signaling pathways [87]. Shi et al. reported that circRNA-NIRP1 is upregulated in OS and competitively binds to miR-532-3p to upregulate Akt3, thus enhancing the activity of the PI3K/Akt pathway and strengthening the malignancy of OS [88]. Hao et al. reported that the overexpression of circRNA-0088214 could inhibit the phosphorylation of Akt to suppress the PI3K/Akt pathway, thereby to cisplatin resistance and promoting apoptosis [89]. Zhang et al. confirmed that hsa-circ-0005909 is upregulated in tumor cells and acts as a molecular sponge for miR-338-3p, reducing its expression compared to that in adjacent cancer tissues. HGMA1, an oncogene that encodes a protein that recognizes and binds continuous A–T base pairs, is involved in gene expression regulation, and is a target of miR-338-3p. The downregulation of miR-338-3p inversely affects the expression of HGMA1. Upregulation of HGMA1 activates the PI3K/Akt pathway, which affects tumor progression [90]. In summary, circRNAs mainly affect tumor cells by sponging miRNAs and sometimes directly influence the phosphorylation of signaling molecules. Further research on this mechanism may provide new insights into the pathogenesis and therapeutic concepts of OS.

### 3.4 Small interfering RNA (siRNAs) in oncology

siRNAs are nucleic acids whose primary role in the body is to degrade target genes via RNA interference. As our understanding of siRNAs deepens, the mechanism of siRNA is thought to be applicable to the treatment of tumors. Hu et al. [91] suggested that siRNAs can silence related oncogenes or cancer-promoting regulatory genes in a sequence-specific manner, thereby reducing the probability of tumor occurrence. Furthermore, studies by Kara et al. [92] proposed that siRNAs can also be used to treat existing cancers or serve as delivery vehicles for certain targeted anti-tumor drugs. Nanotherapy has a vast potential for development. However, surgery combined with adjuvant radiotherapy and chemotherapy remains the primary approach for treating OS. siRNA is a hot field today, and as our understanding deepens, researchers will have updated insights into tumor treatment.

**Figure 1:**
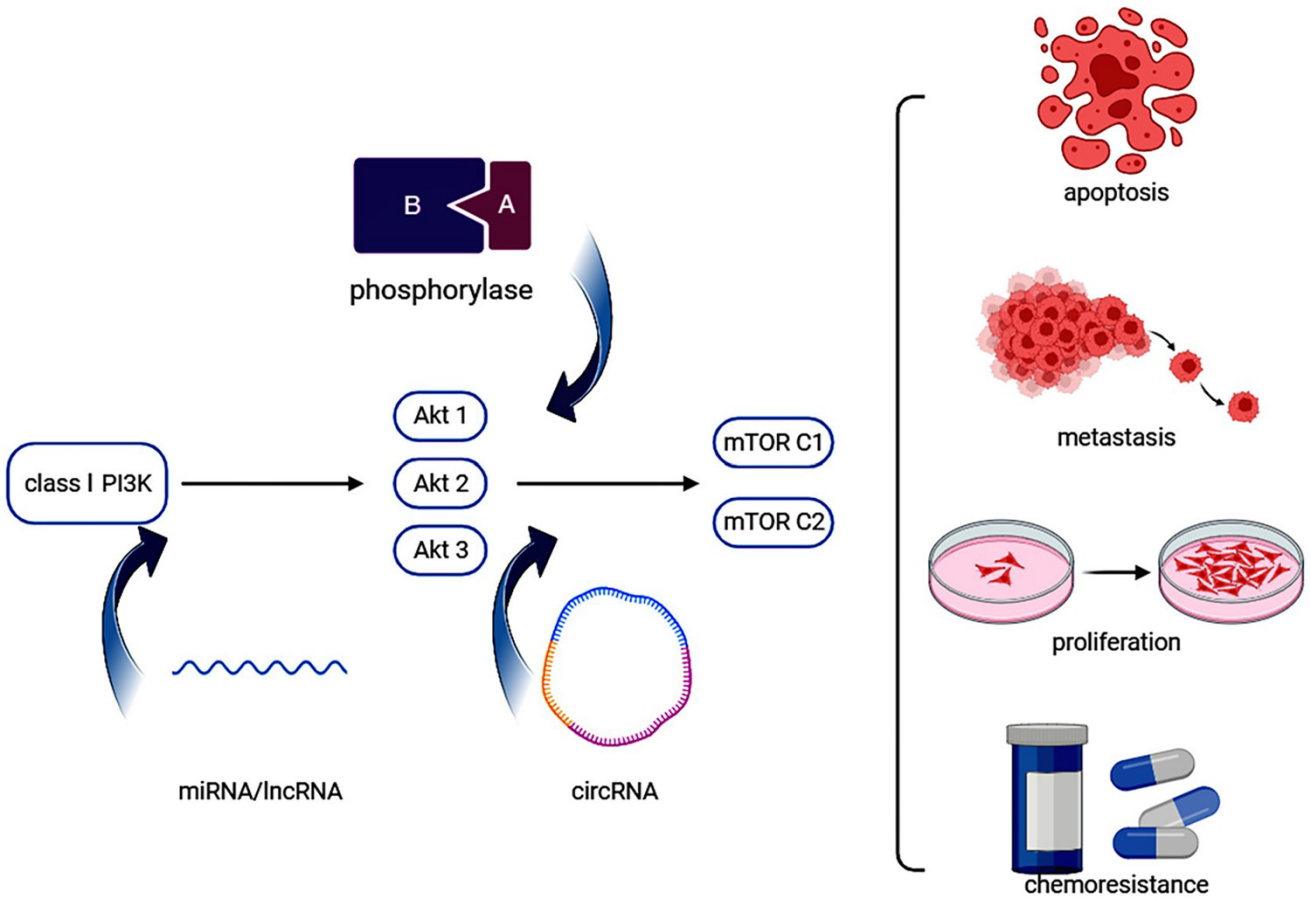
Mechanism of PI3K/Akt/mTOR pathway in OS.

**Figure 2:**
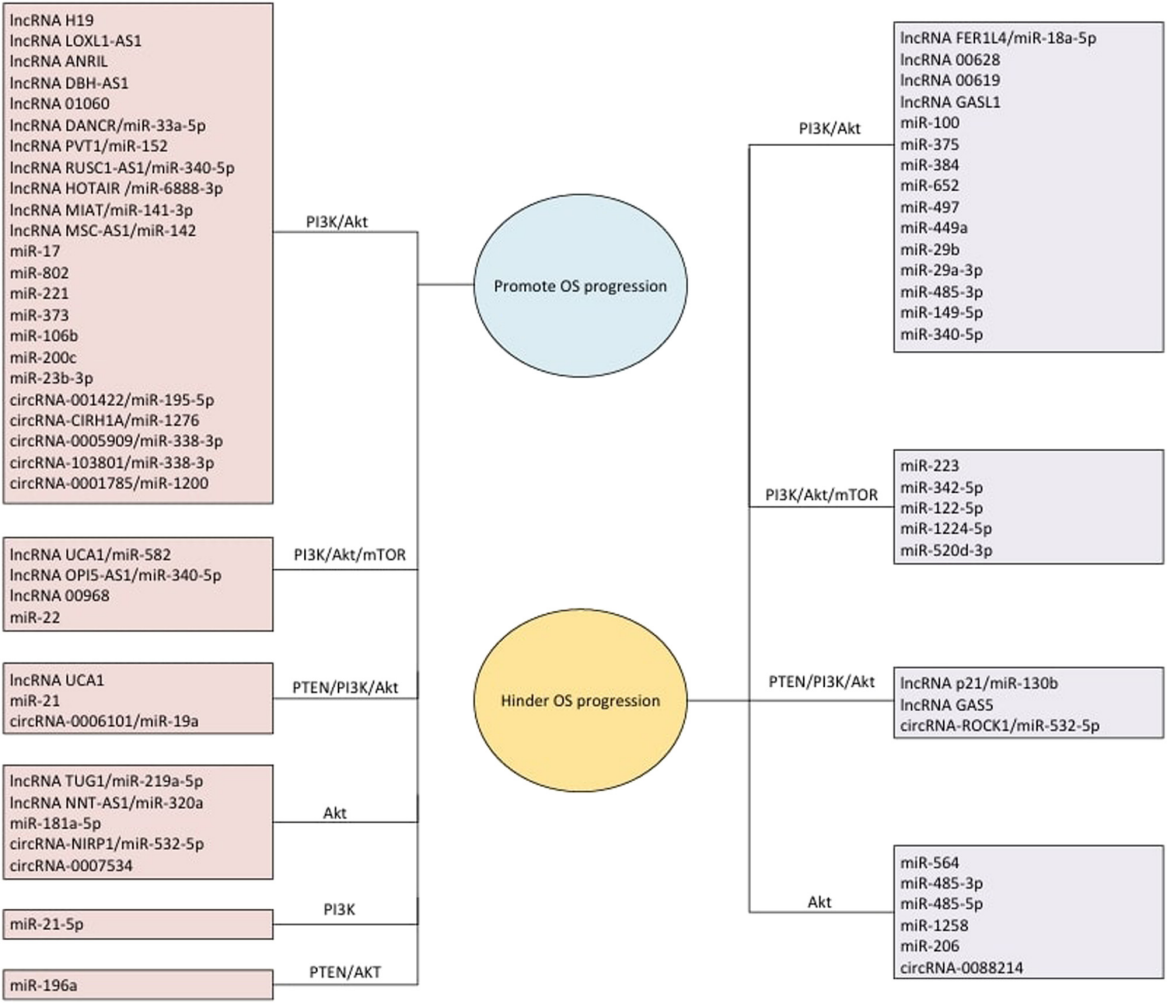
Interactions between the PI3K/AKT/mTOR pathway and ncRNAs in OS.

To provide a more visual representation of the role of the PI3K/Akt/mTOR pathway and ncRNAs in OS, the relationship is summarized. Figure 1 summarizes the impact of the PI3K/Akt/mTOR pathway and ncRNAs on the progression of OS. Three types of ncRNAs exert varying effects on tumor proliferation, metastasis, and other biological behaviors by interacting with the PI3K/Akt/mTOR pathway, ultimately impacting patients with OS on a macroscopic scale. Figure 2 summarizes the roles of various RNAs discussed in this study. Tables 1 and 2 further show the target mechanisms and action proteins of different ncRNAs, compiled from respective referenced literature sources. Figures 1 and 2 were made by the authors using the Biorender (https://app.biorender.com/).

**Table 1:** ncRNAs acted as oncogenes in OS

ncRNAs	Targets	Biological function	Ref.
lncRNA H19	NF-κB pathway	Promotes migration and invasion	[49]
lncRNA MIAT	miR-141-3p/SIX1	Promotes tumor development	[93]
lncRNA HOTAIR	miR-6888-3p/SYK	Promotes the proliferation and migration	[94]
lncRNA 00968	/	Promotes cell survival and colony formation	[50]
lncRNA RUSC1-AS1	miR-340-5p	Promotes the progression and EMT both *in vitro* and *in vivo*	[51]
lncRNA MSC-AS1	miR-142/CDK6	Promotes OS progression and sensitivity to cisplatin	[95]
lncRNA UCA1	HIF-1α/PTEN	Promotes cell growth	[53]
	miR-582/CREB1	Promotes EMT and metastasis	[54]
lncRNA TUG1	miR-219a-5p/FOXM1	Accelerated OS proliferation, migration, and invasion	[55]
lncRNA NNT-AS1	miR-320a	Enhance the continuation of U2 OS and prolong the life span of tumor cells	[57]
lncRNA OPI5-AS1	miR-340-5p/LPAATβ	Causes cisplatin resistance in OS	[61]
lncRNA LOXL1-AS1	/	Promotes cell proliferation, migration, and invasion	[58]
lncRNA DNACR	miR-33a-5p/AXL	Promotes tumor progression and cancer stemness features	[59]
lncRNA ANRIL	/	Accelerates the tumor development	[60]
lncRNA PVT1	miR-152/c-MET	Enhance chemoresistance of OS to gemcitabine	[62]
LncRNA DBH-AS1		Promotes cell proliferation, migration and invasion, and inhibits apoptosis in OS	[96]
LncRNA01060	/	Promotes OS cell malignant behaviors, hyperproliferation, invasion, migration, and EMT	[97]
miR-17	SASH1	Promotes OS cells proliferation, migration, and inhibits apoptosis	[98]
miR-21	PTEN	Promotes proliferation and inhibits apoptosis	[99]
miR-22	PI3K/Akt/mTOR	Enhanced the anti‑proliferative ability of CDDP *in vivo* and *in vitro*	[78]
miR-21-5p	PIK3R1	Promote OS cell proliferation and invasion	[69]
miR-23b-3p	VEPH1	Accelerated the tumor development	[100]
miR-106b		Promote proliferation and invasion	[101]
miR-373	p53	Promotes growth and cellular invasion in OS cells	[102]
miR-802	p27	Promoted the progress of EMT, migration, and invasion	[103]
miR-221	PTEN	Induces cell survival and cisplatin resistance	[104]
miR-196a		Promotes cell proliferation and inhibits cell apoptosis	[105]
miR-200c	CDH1	Promotes the progression and metastasis	[106]
circRNA-ORC2	miR-19a/PTEN	Promotes OS cell growth and invasion	[81]
circRNA-0001785	miR-1200/HOXB2	Promotes proliferative ability, inhibits the apoptosis of OS cells	[83]
circRNA-001422	miR-195-5p/FGF2	Promotes tumor proliferation and metastasis, and inhibits apoptosis	[84]
circRNA-CIRH1A	miR-1276	Promotes the proliferation, invasion, migration	[85]
circRNA-103801	/	Accelerates the development of OS	[86]
	miR-338-3p, HIF-1/Rap1	Accelerates proliferation	[107]
circRNA-0007534	AKT/GSK-3β	Promotes cell growth and inhibits apoptosis	[87]
circRNA-NIRP1	miR-532-3p/PI3K/AKT	Promotes the malignant degree of OS	[88]
circRNA-0005909	miR-338-3p/HGMA1	Promotes the OS progression	[90]

**Table 2:** ncRNAs acted as tumor suppressors in OS

ncRNAs	Targets	Biological function	Ref.
lncRNA p21	miR-130b/PTEN	Inhibits OS cell growth and colony formation	[52]
lncRNA FER1L4	miR-18a-5p	Promotes apoptosis and inhibited the EMT	[56]
	/	Promotes apoptosis and suppresses EMT and stemness markers	[108]
lncRNA GASL1		Inhibits proliferation and invasion of OS cells	[109]
lncRNA GAS5	miR-23a-3p	Suppresses the proliferation and invasion	[110]
lncRNA 00619	HGF	Inhibits proliferation, migration, and invasion and improves apoptosis of OS cells	[111]
lncRNA 00628		Inhibited the proliferation, invasion, and migration and promoted cell apoptosis	[112]
miR-384	SLBP	Inhibits the growth and metastasis	[70]
miR-340-5p	NRF2	Inhibits the malignant phenotypes of OS	[113]
miR-29a-3p	IGF1	Repressed the OS evolvement through inducing autophagy	[71]
miR-29b	Spin 1	Represses OS cell self-renewal, differentiation, and drug resistance	[114]
miR-342-5p	Wnt7b	Inhibits OS cell growth, migration, invasion, and sensitivity to doxorubicin	[72]
miR-485-3p	c-MET, AKT3/mTOR	Inhibits OS glycolysis and metastasis	[73]
	HSP90/AKT1	Suppress OS cell proliferation and migration	[77]
miR-1224-5p	PLK1	Inhibits the proliferation, invasion, and EMT	[74]
miR-564	Akt	Inhibits the proliferation	[75]
miR-149-5p	TWEAK/Fn14	Inhibit the survival and migration of tumor cells	[76]
miR-122-5p	TP53	Inhibits the proliferation and promote the apoptosis of OS cells	[115]
miR-497		Inhibits cell survival and enhanced the sensitivity to cisplatin	[116]
miR-449a	EZH2	Inhibits cell proliferation, invasion, and migration	[117]
miR-520d-3p	MIG-7	Inhibits vasculogenic mimicry formation and metastasis	[118]
miR-100	IGFIR	Inhibits OS cell proliferation, migration, and invasion and enhances chemosensitivity	[119]
miR-206	PAX3/MET	Reduces OS cell malignancy *in vitro*	[120]
miR-375	PIK3CA	Inhibits the tumorigenesis	[121]
miR-146b-5p	TRAF6	Inhibits the proliferation	[122]
miR-223	Hsp90B1	Inhibits the cell growth	[114]
miR-16-5p	TSPAN15	Inhibits the proliferation, migration, and invasion	[123]
circRNA-ROCK1	miR-532-5p/PTEN	Suppresses OS proliferation and migration	[82]
circRNA-0088214	AKT	Suppresses tumor progression	[89]

## 4 Discussion and perspectives

OS is the most common primary malignant tumor of the bone and soft tissue, garnering considerable attention due to its high tumor heterogeneity, poor prognosis, and lung metastasis. Currently, surgery and chemotherapy are the main treatment strategy for patients with OS. However, the lack of targeted drugs is the clinical bottleneck of the treatment for the patients with OS. Thus, there is a need urgent to study the pathogenesis and metastasis of OS. As one of the important signal transduction pathways in tumor cells, PI3K/AKT/mTOR signaling pathway plays an important role in the development and drug sensitivity of OS, with functions such as accelerating cell cycle, promoting cell proliferation, invasion and metastasis, and enhancing drug sensitivity.

Increasing evidence suggests that ncRNAs can regulate the PI3K/Akt/mTOR signaling pathway in OS. This pathway influences tumor progression and may exacerbate chemotherapy resistance. Numerous ncRNAs in OS tissues have the capability of promoting or inhibiting the activation of the PI3K/Akt/mTOR pathway. This study reviews the interactions between various ncRNAs and the PI3K/Akt/mTOR pathway and discovers new applications of ncRNAs based on their effects on this pathway. We categorize ncRNAs that regulate signaling pathways into two types. Some RNAs are upregulated in OS tissues, indicating their oncogenic potential. These ncRNAs activate the PI3K/Akt/mTOR pathway, promoting cancer cell proliferation, invasion, inhibiting apoptosis, and enhancing drug resistance. Conversely, other ncRNAs are downregulated in OS tissues, exerting opposite effects to inhibit tumor progression. Therefore, we can utilize these molecular mechanisms to predict the prognosis of OS by measuring the expression levels and trends of RNA or PI3K/Akt pathway molecules *in vivo*. As described, taking lncRNA LOXL1-AS1 as an example, its upregulation in OS tissues has oncogenic effects. Thus, clinically measuring the expression level of lncRNA LOXL1-AS1, if found to be elevated above normal levels, indicates a potentially poorer prognosis for the patient. With further research, more ncRNAs with similar functions will likely be discovered, expanding our options for diagnosing diseases and predicting prognosis more accurately.

Many key proteins in the PI3K/AKT/mTOR signaling pathway are also important targets for drug design, and the study of small molecule inhibitors has also made great progress. Many drug candidates have entered clinical studies, including PI3K inhibitors, AKT inhibitors, and dual PI3K/mTOR inhibitors. Researchers have proposed a nanocarrier for targeted delivery of ncRNAs for gene therapy. This nanocarrier exhibits low cytotoxicity and effective intracellular transmission in tumor cells. For example, the results of some researchers suggest that delivery of miR-22 targeting the PI3K/Akt pathway has good anti-tumor activity [124]. However, applying theoretical concepts to clinical practice faces several challenges. First, RNA expression involves complex mechanisms and whether clinical interventions can adjust RNA expression to affect pathway activation remains unclear. Second, each patient with OS may have different genes with abnormal expression genes, posing difficulties in whether such carriers can deliver other types of RNAs and how therapeutic nucleic acids can be obtained. Third, the cost-effectiveness of this treatment method is a concern. Whether costs can be controlled and reduced determines its feasibility for widespread implementation globally, which needs careful consideration.

Therefore, further understanding of the function and molecular mechanism of the interaction of PI3K/AKT/mTOR signaling pathway and ncRNAs is of great significance for finding new therapeutic approaches and potential targets for OS, and ultimately improving the prognosis of patients with OS.
